# Transforming Perceptions: Exploring the Multifaceted Potential of Generative AI for People With Cognitive Disabilities

**DOI:** 10.2196/64182

**Published:** 2025-01-15

**Authors:** Dorit Hadar Souval, Yuval Haber, Amir Tal, Tomer Simon, Tal Elyoseph, Zohar Elyoseph

**Affiliations:** 1 Max Stern Academic College of Emek Yezreel, Tel Adashim, Israel; 2 Interdisciplinary Studies Unit, PhD Program of Hermeneutics and Cultural Studies, Bar-Ilan University, Ramat Gan, Israel; 3 Tel Aviv University, Tel Aviv, Israel; 4 Microsoft Israel R&D Center, Tel Aviv, Israel; 5 Faculty of Social Welfare and Health Sciences, University of Haifa, Haifa, Israel; 6 Imperial College London, Fulham Palace Rd, London, W6 8RF, United Kingdom, 44 547836088; 7 Faculty of Education, University of Haifa, Haifa, Israel

**Keywords:** generative artificial intelligence, cognitive disability, social participation, AI ethics, assistive technology, cognitive disorder, societal barriers, social inclusion, disability study, social mirror, cognitive partner, empowerment, user involvement, GenAI, artificial intelligence, neurotechnology, neuroinformatics, digital health, health informatics, neuroscience, mental health, computer science, machine learning

## Abstract

**Background:**

The emergence of generative artificial intelligence (GenAI) presents unprecedented opportunities to redefine conceptions of personhood and cognitive disability, potentially enhancing the inclusion and participation of individuals with cognitive disabilities in society.

**Objective:**

We aim to explore the transformative potential of GenAI in reshaping perceptions of cognitive disability, dismantling societal barriers, and promoting social participation for individuals with cognitive disabilities.

**Methods:**

This study is a critical review of current literature in disability studies, artificial intelligence (AI) ethics, and computer science, integrating insights from disability theories and the philosophy of technology. The analysis focused on 2 key aspects: GenAI as a social mirror reflecting societal values and biases, and GenAI as a cognitive partner for individuals with cognitive disabilities.

**Results:**

This paper proposes a theoretical framework for understanding the impact of GenAI on perceptions of cognitive disability. It introduces the concepts of GenAI as a “social mirror” that reflects and potentially amplifies societal biases and as a “cognitive copilot” providing personalized assistance in daily tasks, social interactions, and environmental navigation. This paper also presents a novel protocol for developing AI systems tailored to the needs of individuals with cognitive disabilities, emphasizing user involvement, ethical considerations, and the need to address both the opportunities and challenges posed by GenAI.

**Conclusions:**

Although GenAI has great potential for promoting the inclusion and empowerment of individuals with cognitive disabilities, realizing this potential requires a change in societal attitudes and development practices. This paper calls for interdisciplinary collaboration and close partnership with the disability community in the development and implementation of GenAI technologies. Realizing the potential of GenAI for promoting the inclusion and empowerment of individuals with cognitive disabilities requires a multifaceted approach. This involves a shift in societal attitudes, inclusive AI development practices that prioritize the needs and perspectives of the disability community, and ongoing interdisciplinary collaboration. This paper emphasizes the importance of proceeding with caution, recognizing the ethical complexities and potential risks alongside the transformative possibilities of GenAI technology.

## Introduction

In the era of generative artificial intelligence (GenAI), traditional notions of personhood and normality are being challenged [1-4]. Technological advances are blurring the boundaries between human and machine capabilities, offering an opportunity to expand the limits of social inclusion and promote change in attitudes toward people with disabilities [1]. As artificial intelligence (AI) systems demonstrate increasingly sophisticated cognitive abilities, they prompt us to reconsider what qualities define personhood and human intelligence. This paper examines the potential of GenAI to disrupt limiting conceptions of morality and humanity, focusing on the implications of GenAI for the social status of people with cognitive disabilities. This paper also proposes a practical toolkit for GenAI development and engineering professionals—product managers, data scientists, and developers—to help incorporate these insights into their work.

Cognitive disability refers to a wide range of impairments affecting cognitive functions such as learning, problem-solving, judgment, communication, and social interaction [5]. Examples of cognitive disabilities include intellectual disability, attention-deficit/hyperactivity disorder, autism spectrum disorders, specific learning disabilities (such as dyslexia), and brain injuries (such as traumatic brain injury or stroke) [5-7]. It is important to emphasize the variety of individuals with cognitive disabilities, each one possessing a unique combination of strengths, impairments, and potential, which means that cognitive disabilities require personalized approaches to intervention. While recognizing the diverse nature of cognitive disabilities and the need for tailored solutions, this paper focuses on the general potential of GenAI to improve the lives of people across the spectrum of cognitive disabilities.

Engaging with the integration of GenAI and individuals with cognitive disabilities is a new direction in the use of technology in the field of disability. The potential for AI to support and empower this population lies in its ability to perform cognitive tasks such as reasoning, planning, decision-making, and communication—areas that are challenging for people with cognitive disabilities [8-10]. The ability of AI to remove barriers and open new paths for inclusive and equitable participation makes it especially relevant for this population [11]. An in-depth analysis of this ability requires examining the philosophical and ethical implications of AI for conceptions of humanity and morality, questions that directly determine how society views and accommodates individuals with cognitive disabilities. These are fundamental inquiries into the nature of intelligence, personhood, consciousness, and human agency, which largely determine the degree of participation and inclusion for this group.

## Personhood and AI: An Opportunity for Paradigm Shift

The concept of personhood, which emerged as a central topic in bioethical debates surrounding topics such as abortion, stem cell research, and euthanasia, has evolved into a complex and multifaceted construct that now spans multiple disciplines [12]. Inherently normative in nature, personhood involves value judgments and ethical considerations regarding how we ought to treat and perceive others rather than merely describing observable facts. Personhood is not rooted exclusively in our biology and experiences but in our essence and identity. This identity, however, is not formed in isolation; it is dynamically shaped in an intricate interaction between self-perception and the perception of others and interaction with them. Rosfort [13] argued that this conceptualization of personhood reveals its profoundly relational and social nature, demonstrating how identity and perception of self-worth are inextricably woven into interactions and the broader human context.

The concept of “personhood” has long served as a central criterion in bioethical discussions, determining which entities deserve moral consideration and rights [3]. As a result, this notion has also functioned as a mechanism of exclusion, denying basic rights and opportunities to those deemed cognitively “abnormal” [14].

For example, historically, people with cognitive disabilities were excluded from the public sphere and denied the right to make decisions for themselves [15,16]. Even today, despite significant progress in discourse and work based on the “social model” (an approach that views disability as created by societal barriers rather than by individual impairments alone) [17] and the “minority group model” (which recognizes people with disabilities as a marginalized minority group) [18], exclusion still exists in various aspects of life. People with cognitive disabilities still face barriers to accessing higher education and vocational training because of preconceived notions about their abilities [19]. Despite relevant skills, they have difficulties securing meaningful employment and career advancement opportunities because of social stigma and prejudice [20]. Participation in political or civic decision-making processes, such as voting or community involvement, is limited by discriminatory perceptions of the competence of individuals with cognitive disabilities [21]. They are also excluded from leisure, social, and cultural activities because of a lack of access or restrictive attitudes toward their participation [22].

These exclusion examples illustrate how, as a result of conceptualizing what constitutes a person of merit, individuals with cognitive disabilities are often excluded in the deepest and broadest ways from society. This mechanism is difficult to identify because it operates through our language and the most basic organized mechanisms of any society: law, health care system, education system, and more [23].

Breaking entrenched concepts and perceptions of personhood is challenging because they are deeply embedded in societal structures and norms, but emerging technologies are beginning to challenge these long held beliefs. GenAI offers an opportunity to challenge the definition of personhood perceptions by demonstrating skills previously considered unique to humans [1,4]. Although these capabilities are not yet perfect in AI, their very existence challenges the idea that such traits belong exclusively to the “normal” cognitive function of humans and that social participation is conditional on the presence of these abilities.

The revolutionary potential of GenAI invites us to reexamine the criteria for membership in the moral community and expand them beyond limiting standards. Instead of relying on a narrow model of “correct” cognitive abilities as a prerequisite for rights and participation in society [14], we may adopt, with the assistance of GenAI, a more inclusive view that recognizes human diversity and the inherent value of all individuals, regardless of their abilities [24]. By showcasing the potential of machines to exhibit complex cognitive traits, GenAI challenges the notion that certain abilities are essential for personhood and moral status. It initiates a discourse on the need to redefine our understanding of what it means to be human and to have moral worth, moving away from a focus on cognitive benchmarks and toward a more encompassing vision of human dignity and rights [1,4].

Although AI presents opportunities to challenge our understanding of personhood, there are legitimate concerns about its potential to exacerbate exclusion and narrow definitions of “normal” human cognition. The inherent biases in AI systems, stemming from their training data and algorithmic design [25-28], risk reinforcing and amplifying existing societal prejudices [29]. As AI increasingly influences decision-making processes in areas such as employment, health care, and criminal justice, there is a danger that it could lead to more stringent and narrow criteria for what constitutes “normal” human functioning. This could inadvertently heighten barriers for individuals with cognitive differences, further marginalizing them from full societal participation [30]. Moreover, as AI systems become more sophisticated in mimicking certain human cognitive abilities, there is a risk that societal expectations of human performance might be unrealistically elevated, potentially creating an even more exclusionary standard of “normal” [31]. Thus, while AI challenges our notions of personhood, it simultaneously risks entrenching and exacerbating existing forms of exclusion, highlighting the critical need for ethical AI development and deployment considering diverse human experiences and capabilities. In the following sections, we will explore 2 key areas where GenAI has the potential to drive significant change: GenAI as a social mirror and GenAI as a cognitive partner. These 2 domains highlight the multifaceted impact that GenAI can have on reshaping perceptions, removing barriers, and promoting participation of individuals with cognitive disabilities on the one hand, and exacerbating existing biases and exclusions in society on the other.

## Generative AI as a Social Mirror: Opportunity and Challenge

### Overview

Vallor’s [32] conceptualization of AI as a societal mirror provides a compelling framework for understanding the role of AI in reflecting and potentially amplifying societal biases, particularly concerning cognitive disabilities. This mirror metaphor can be understood as follows: just as a physical mirror reflects the image of what stands before it, AI systems reflect the data, values, and biases present in the society that created them. However, unlike a simple reflection, AI systems can amplify and distort these reflections, much as a funhouse mirror might exaggerate certain features.

This mirror effect illuminates how AI systems, trained on biased data, risk perpetuating existing prejudices against individuals with cognitive differences. AI essentially learns from and then projects back the biases inherent in its training data, potentially reinforcing and spreading these biases further. Paradoxically, this same reflective quality presents a unique opportunity to identify and address longstanding societal biases, rendering implicit prejudices explicit and subject to scrutiny. By closely examining what the AI “reflects back” to us, we can gain insights into biases that might otherwise remain hidden or unacknowledged in society.

Vallor [32] posits that AI systems in general, and GenAI systems in particular, are not merely neutral technological tools but mirrors reflecting the values, norms, and biases prevalent in human society. Given that these systems are constructed upon data and content created by humans, they inherently risk replicating and perpetuating prejudices and discrimination against marginalized groups, including people with cognitive disabilities [27,33].

A study by Gadiraju et al [34] demonstrated this mirroring effect in action. They conducted 19 focus groups with 56 participants with various disabilities who interacted with a dialog model based on a large language model. The researchers found that the model frequently perpetuated harmful stereotypes and narratives about disability. For example, the model often fixated on physical disabilities, particularly wheelchairs, while neglecting other types of disabilities. It also tended to portray people with disabilities as passive, sad, and lonely, reinforcing the misconception that disability is inherently negative. Additionally, the model sometimes produced what participants referred to as “inspiration porn,” objectifying people with disabilities as sources of inspiration for nondisabled people.

For example, if the information used to train AI systems contains stereotypical or derogatory expressions toward people with cognitive disabilities, there is a significant risk that these systems might “learn” to adopt discriminatory attitudes. The potential consequences are severe: AI systems could rank individuals with cognitive disabilities as having lower potential in employment or educational contexts, limit their access to certain services, or make biased decisions about them in critical areas such as insurance or credit [35].

When we look into the societal mirror reflected by AI, several possible human responses can be identified. One metaphorical response is “breaking the mirror,” representing human resistance to AI use and the insights it presents [36]. While this approach attempts to avoid the uncomfortable truths AI exposes, it risks missing out on the potential benefits and insights AI can offer. Another metaphorical strategy is “cleaning the mirror,” where humans attempt to eliminate biases through AI alignment processes [37]. This approach aims to create AI systems aligned with human values and intentions, striving for a bias-free environment. However, it risks producing an artificially sterile system that fails to reflect the complexities of human cognition and interaction, potentially making AI less relevant and less capable of addressing real-world complexities.

The third and most promising approach involves using reflection as a call to action in the real world. This method requires humans to acknowledge the biases reflected by AI and use this awareness as a catalyst for societal change. It demands active engagement and concrete actions from us as humans to address these issues, both in our AI systems and in society at large [38]. This approach recognizes that if such action is taken, over time, the reflection in the AI mirror itself can change, not as a result of erasing biases in the machine as in the second option, but as a consequence of real societal change that is then differently reflected in the AI mirror.

To implement this approach specifically within the realm of AI development and deployment, we must adopt advanced techniques and ensure inclusive human involvement. As contemporary AI systems increasingly incorporate vast datasets populated from the internet, traditional methods of addressing biases through direct data manipulation, such as the “datasheets” approach proposed by Gebru et al [39], while still valuable in certain contexts, have become more challenging to implement comprehensively. This shift has led to the adoption of complementary techniques that can handle the scale and complexity of modern AI systems such as self-supervised learning [40] and reward modeling [41]. Crucially, these techniques still require human decision-making at key junctures. To truly address biases and create more equitable AI systems, particularly regarding cognitive disabilities, we must ensure that people with cognitive disabilities are actively involved in these decision-making processes. This collaborative approach aligns with our third strategy, emphasizing real-world action and societal change. By critically examining the biases revealed in AI outputs and involving diverse perspectives in the development process, we can work toward creating more inclusive AI systems. This approach not only helps in developing fairer algorithms and more representative models but also contributes to broader societal change [1,4]. In this way, the AI mirror becomes not just a reflection of our current culture, but a catalyst for the more inclusive society we aspire to create [16,42].

**Figure 1.**
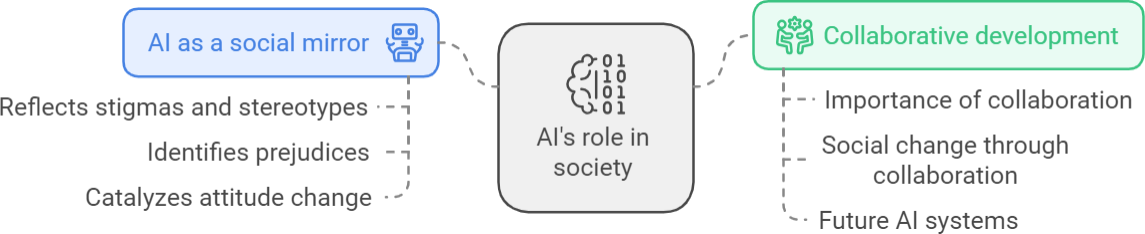
GenAI as a social mirror: collaborative development for societal change. AI: artificial intelligence; GenAI: generative artificial intelligence.

In conclusion, as illustrated in Figure 1, GenAI has the potential to promote social justice and shift perceptions regarding cognitive disabilities. To harness this potential, collaborative work and ongoing effort are required to embed values of accessibility, inclusion, and respect for diversity at the core of technological development. These steps can transform the “reflection in the mirror” into a positive and inclusive image for people with cognitive disabilities, potentially leading to broader societal changes in perception and inclusion.

While this mirror metaphor provides valuable insights, it is important to recognize its limitations. Vallor’s conceptualization, though powerful, doesn’t fully capture the multifaceted potential of AI, particularly for people with disabilities. It overlooks its capability to actively solve previously intractable problems and enhance accessibility. To provide a more comprehensive understanding, we must expand our view beyond the perception of AI as a mere reflective tool. In the following section, we propose considering AI not only as a mirror but also as a cognitive partner for people with disabilities, emphasizing its potential to actively support and empower individuals with cognitive differences in navigating the world.

### Generative AI as a Cognitive Partner for People With Disabilities

Beyond Vallor’s mirror metaphor for AI and its contingent inference on social change for people with cognitive disabilities, a significant potential of GenAI lies in its ability to serve as a “cognitive partner,” empowering participation of these people in life domains that were previously blocked or limited for them [43-45]. This partnership can be metaphorically described as a “cognitive copilot” (an AI assistant for complex cognitive tasks), assisting and empowering the individual with tasks requiring complex cognitive functions. For example, GenAI can help a person with cognitive disabilities manage daily tasks such as scheduling, budgeting, or navigating urban spaces by providing personalized reminders, recommendations, and guidance [46,47]. Additionally, it can serve as an advisor in complex social situations, such as interpreting body language [48], suggesting appropriate responses to expressions of anger or mockery from others, or assisting in decision-making [1,49]. In this way, GenAI may act as a kind of “social copilot,” providing real-time support and feedback, allowing persons with cognitive disability to expand their circle of social interactions, inclusion, and activities.

One of the outstanding strengths of GenAI is its ability to function as a translator and mediator between languages, concepts, and realities. For people with cognitive disabilities, translation and mediation pose a central challenge in daily life, both in understanding the environment and in expressing themselves in a way others can understand [50]. With its natural learning and processing capabilities, GenAI can bridge these gaps and make information and communication more accessible.

**Figure 2.**
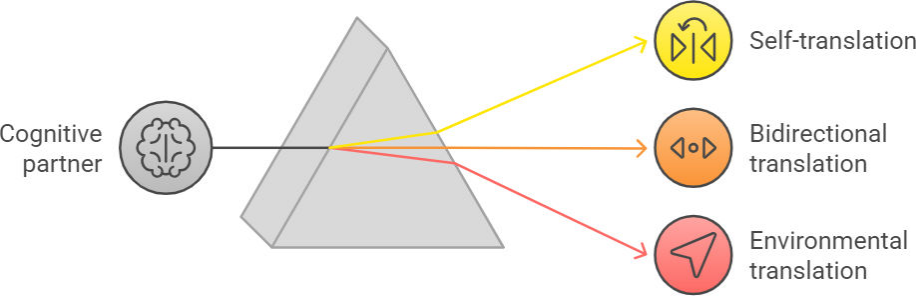
Three main areas of GenAI application as a cognitive partner. GenAI: generative artificial intelligence.

The application of GenAI as a cognitive copilot can focus on 3 main areas (Figure 2):

1. Translating and making the inner world of people with cognitive disability accessible to themselves: GenAI can help people with cognitive disabilities better understand themselves, their thoughts, emotions, and needs. This is achieved by providing explanations and conceptualizations in clear and accessible language, identifying and interpreting emotional states, and suggesting strategies for coping with challenges [50]. GenAI can serve as an “internal translator” that through a process of assistive conceptual scaffolding and cognitive structuring [51] assists individuals in accurate self-understanding and self-expression.
2. Bidirectional translation and mediation in interpersonal communication: By analyzing interpersonal and social information, GenAI can mediate interactions with other people, making it possible to negotiate the complexities inherent in human communication more successfully. The unique contribution of GenAI in this area lies in its ability to bridge the communication gap in both directions, helping the person with cognitive disability understand the social environment, the intentions of others, and the implicit messages in discourse, and making the person’s wants, needs, and emotions more accessible to the social environment [1]. For example, on one hand, GenAI can offer interpretations of social cues and recommend appropriate responses, and on the other assist individuals in articulating their thoughts more clearly and presenting their unique perspectives. The technology can serve as a “two-way social translator,” enabling people with disability and their environment to better understand each other and promote respectful and equitable communication.
3. Making the physical environment and public spaces accessible: GenAI can act as an “environmental translator,” converting complex information about the world into a clear and disability-friendly format. This can include, for example, simplifying official texts, graphically converting numeric data, or creating interactive guides for navigating public spaces [52]. Thus, GenAI models that are open to the public can “see” and “understand” photos and videos and describe their content [1], so that people with cognitive disabilities may gain greater access and independence in managing their lives.

The goal is not to “normalize” individuals with cognitive disabilities or to erase their disability. The cognitive partner metaphor, similar to Vallor’s mirror metaphor, can show how the use of AI might exacerbate exclusionary attitudes and further marginalize individuals with disabilities. Therefore, using AI for social change in our attitude toward people with cognitive disabilities means that the aim of this technology should be to enable access to environments and spaces that were previously closed or socially inaccessible to them, while also facilitating the accessibility of these environments to the individuals themselves. The approach should be person-centered, respecting diversity, and tailored to the unique aspirations and needs of everyone, rather than imposing a uniform standard of “proper” functioning.

Serious consideration must be given to the ethical implications of such a close integration between humans and machines, particularly in the areas of autonomy and responsibility. Questions of privacy, data security, and people’s ownership of decisions made by AI systems need to be thoroughly examined [52,53]. Robust oversight and regulatory mechanisms must be in place to ensure the responsible and ethical use of AI, safeguarding the rights and well-being of users. This is especially critical when working with vulnerable populations such as people with cognitive disabilities, where protecting individual autonomy is important [27,33].

In conclusion, although AI-based “cognitive copilot” applications for people with cognitive disabilities have the potential to remove barriers, increase participation, and promote equal opportunities across various domains of life, it is essential to proceed with caution. This technology must function as a “translator” to contribute to a more inclusive and equitable society, and we must remain vigilant to its risks. Ensuring that AI development is person-centered, ethically sound, and involves active participation from the disability community is crucial for harnessing its benefits without worsening existing biases and systemic barriers.

### Implication for AI Developers and Technologists

GenAI has immense potential to promote inclusion and equality for people with cognitive disabilities but to realize this potential requires a perceptual shift on the part of developers, engineers, researchers, and product managers. Instead of focusing narrowly on “fixing” certain impairments, they must adopt a more holistic approach that views technology as a lever for social integration and broad improvement in quality of life [54-56]. This involves a transition from regarding GenAI as a mere technical solution to perceiving it as a tool for effecting social change for the population with cognitive disabilities.

In practice, close and ongoing collaboration with people with cognitive disabilities throughout all stages of development is important [57]. Development teams must learn from the unique experiences and needs of individuals with cognitive disability and meaningfully integrate them into the design and construction of GenAI systems and prompts.

Recent research has demonstrated the feasibility and importance of this approach. For example, Newbutt et al [58] conducted a systematic review of studies involving autistic individuals in the design of extended reality technologies. They found that out of 20 studies published between 2002‐2022, several successfully engaged autistic individuals as active co-designers and cocreators, allowing them to shape the final products according to their needs and preferences. This highlights the growing trend and importance of including the target users in the design process.

This requires a joint definition of goals, adapting user interfaces and user experience to their modes of thinking and communication, and clearly formulating principles of cognitive accessibility from the earliest planning stages [59]. The aspiration is for the empowerment and inclusion of people with cognitive disabilities to be embedded in the core of the technology and in the layer of its use.

Bircanin et al [60] presented a practical approach to including adults with severe intellectual disabilities in co-design through active support. They demonstrated how principles such as “every moment has potential,” “graded assistance,” “little and often,” and “maximizing choice and control” can be applied in design contexts to ensure meaningful participation of individuals with severe cognitive disabilities. This approach provides concrete strategies for AI developers to engage with this population during the development process.

For example, it is important to examine how the prompt-based user interface can be made accessible and adapted to the cognitive and communication characteristics of people with different types of cognitive disabilities. Consideration should be given to whether the development of dedicated products is the right direction or whether personal adaptation at the level of the individual user is preferable [61]. Answering such questions requires ongoing discourse and feedback from the community itself.

Dirks [57] explored the ethical challenges in inclusive software development projects with people with cognitive disabilities. The study emphasized the importance of maximizing choice and control for participants, using a graded assistance approach, and ensuring every moment has potential for meaningful engagement. These principles can guide AI developers in creating more inclusive design processes.

To assist developers and researchers in implementing the principles presented in this paper, we propose a working protocol specifically tailored to the development challenges of GenAI technologies aimed at people with cognitive disabilities. The protocol (Table 1) is based on the model developed by Amershi et al [62], which was formulated following comprehensive research, including a review of academic and industry literature, interviews with experts, and an examination of a wide range of AI-based products. The original model defines 18 general guidelines for designing human-AI interactions across different time frames and stages of interaction. In practice, these guidelines serve as a framework for developing human-centered AI systems, focusing on aspects such as transparency, fairness, reliability, safety, privacy, security, and accountability. Developers and designers use these guidelines to enhance human-AI interaction by implementing practices such as explaining AI decisions to users, designing interfaces that enable user control and feedback, and incorporating mechanisms to identify and mitigate biases [63].

**Table 1.** Protocol for designing artifical intelligence (AI) interactions for people with cognitive disabilities.^a^

Stage and dimension		Guidelines for AI interaction with people with cognitive disabilities	Implementation examples
**Initial**			
	Personal	I1. Identify and adapt to the user’s unique cognitive and emotional needs.	I1. Create a personal profile including preferences, abilities, and challenges.
	Interpersonal	I2. Show awareness of the social and cultural context of system use.	I2. Consider the human environment (eg, caregivers or family members) as part of system definition.
**During interaction**			
	Personal	D1. Provide custom-tailored, gradual, and structured responses to personal needs during use.	D1. Identify difficulties and adapt the level of assistance and feedback in real time.
	Interpersonal	D2. Promote positive and reciprocal communication with the human environment.	D2. Mediate social interactions by simplifying and explaining social cues.
	Environmental	D3. Assist in orientation, navigation, and independent functioning in complex spaces.	D3. Provide detailed instructions and cues on proper conduct in different places.
**When the system errs**			
	Personal	E1. Handle errors respectfully and in an empowering way, with emphasis on learning and progress.	E1. Provide repeated opportunities to try again, together with verbal encouragement.
	Interpersonal	E2. Involve support persons in the process of learning and correction.	E2. Provide a possibility for a caregiver to assist in problem-solving or making necessary adjustments.
	Environmental	E3. Avoid placing responsibility on the user in complex or unexpected situations.	E3. Make human backup available by default in case of significant problems.
**Over time**			
	Personal	T1. Continually adapt to the pace of development, learning, and changes in personal needs.	T1. Track progress and adapt tasks and goals accordingly.
	Interpersonal	T2. Show sensitivity to changes in relationships and roles within the support circle.	T2. Update user profiles and access settings based on feedback from the environment.
	Environmental	T3. Show flexibility and adaptability to changing environments and transitions between contexts.	T3. Automatically detect location changes and provide relevant recommendations.
	Collaboration	T4. Actively involve users and stakeholders in the ongoing development of the system.	T4. Provide mechanisms for receiving feedback and involving users in decisions about updates and improvements.

a The model for this protocol by Amershi et al [62] is based on extensive research and analysis of a range of artificial intelligence products and defines 18 general guidelines across different stages of interaction. We adapted and extended this model to address specifically the needs and challenges of designing artificial intelligence technologies for people with cognitive disabilities. The protocol incorporates 4 key dimensions: personal, interpersonal, environmental, and collaborative, and provides concrete examples of how these considerations can be integrated throughout the life cycle of the artificial intelligence system. By implementing this protocol, developers can create artificial intelligence tools that empower and enhance the lives of individuals with cognitive disabilities.

Building on the analysis presented in this paper, we expand the model of Amershi et al [62] and adapt it to the 4 central dimensions in which AI systems can assist people with cognitive disabilities: the personal, the interpersonal, the environmental, and the collaborative. For each of these dimensions, we propose guidelines and offer practical examples of how the relevant considerations can be embedded at different stages of the system life cycle, from defining the initial requirements, through ongoing interaction, to continuous adaptation and improvement. The proposed protocol serves as a foundation that requires further development, testing, and investigation, but it can serve as a starting point for discourse and the advancement of best practices in designing AI systems for individuals with cognitive disabilities.

## Conclusion

The emergence of GenAI technologies represents a pivotal moment in reconceptualizing disability and personhood. We suggest that the advent of GenAI challenges assumptions about what qualifies an individual as a “person” and questions the notion that cognitive abilities are the sole determinant of one’s rights and societal participation.

In this paper, we explored the transformative potential of GenAI in reshaping perceptions, dismantling barriers, and empowering individuals with cognitive disabilities. By serving as a social mirror [32], AI systems can expose and challenge deeply ingrained biases and prejudices, compelling us to confront the ways we have historically marginalized and excluded the population with cognitive disabilities. Simultaneously, by functioning as a cognitive partner, GenAI may provide unprecedented opportunities for individuals with cognitive disabilities to participate in society.

Realizing this vision requires more than technological innovation, however. It demands a gradual shift in societal attitudes and a sincere effort to involve people with cognitive disabilities in the AI development process, granting them autonomy and recognizing and valuing their abilities. This is where the role of technology professionals and GenAI developers becomes crucial.

The importance of designing AI thoughtfully lies in the understanding that whether we consider AI as a mirror or as a cognitive partner, both metaphors indicate that AI will increasingly mediate how we perceive the world, ourselves, and others, confirming once again McLuhan’s [64] statement that “the medium is the message.” This means that the significant effect of AI lies not merely in the content we explore through it but in how its very use changes us. Therefore, the design and development of AI tools will profoundly influence the future of human society, how we perceive individuals with disabilities, as well as the rights and social positions they will attain. Therefore, how AI is being shaped now will determine its role in reinforcing existing biases or promoting a more inclusive and equitable society.

The proposed protocol, based on the work by Amershi et al [62], offers a practical framework for implementing these principles as part of GenAI development for people with cognitive disabilities. This paper marks only the beginning of the discussion about GenAI and developmental disabilities, therefore we must remain vigilant regarding the ethical and social implications of GenAI and continue to engage in open, multidisciplinary dialogue about how to harness its potential for the greater good.

The path ahead is complex and challenging, but it is also filled with immense possibilities. As we look toward the future, the evolution of AI from reactive, prompt-based systems to proactive, autopilot models promises to further expand these possibilities, particularly for individuals with cognitive disabilities. These advanced systems, capable of learning user needs and initiating interactions without explicit prompts, could provide more seamless and intuitive support, potentially revolutionizing the way we approach cognitive assistance.

Technological progress also involves an ongoing need for ethical and inclusive development. We must prioritize user autonomy and privacy while maximizing the benefits of technological assistance. This balance is important not only for protecting individual rights but also for ensuring that AI serves the needs of those it aims to support.

By embracing the potential of GenAI while remaining vigilant regarding its ethical implications, researchers, developers, and policy makers can create technologies that not only uplift those who have been historically marginalized but enrich the human experience for us all. In doing so, we may take a step toward a future where technology serves as a platform for inclusivity and empowerment.

## Abbreviations

AI: artificial intelligence
GenAI: generative artificial intelligence
